# Whole blood transfusion improves vascular integrity and increases survival in artemether-treated experimental cerebral malaria

**DOI:** 10.1038/s41598-021-91499-3

**Published:** 2021-06-08

**Authors:** Saba Gul, Flavia L. Ribeiro-Gomes, Aline S. Moreira, Guilherme S. Sanches, Fabiana G. Conceição, Cláudio Tadeu Daniel-Ribeiro, Hans C. Ackerman, Leonardo J. M. Carvalho

**Affiliations:** 1 Laboratory of Malaria Research, Oswaldo Cruz Institute, Fiocruz, Av. Brasil 4365, Manguinhos, Rio de Janeiro, 21040-360 Brazil; 2 Laboratory of Malaria and Vector Research, NIAID, NIH, Rockville, MD USA

**Keywords:** Neuro-vascular interactions, Infection, Malaria, Experimental models of disease

## Abstract

Pathological features observed in both human and experimental cerebral malaria (ECM) are endothelial dysfunction and changes in blood components. Blood transfusion has been routinely used in patients with severe malarial anemia and can also benefit comatose and acidotic malaria patients. In the present study *Plasmodium berghei*-infected mice were transfused intraperitoneally with 200 μL of whole blood along with 20 mg/kg of artemether. ECM mice showed severe thrombocytopenia and decreases in hematocrit. Artemether treatment markedly aggravated anemia within 24 h. Whole blood administration significantly prevented further drop in hematocrit and partially restored the platelet count. Increased levels of plasma angiopoietin-2 (Ang-2) remained high 24 h after artemether treatment but returned to normal levels 24 h after blood transfusion, indicating reversal to quiescence. Ang-1 was depleted in ECM mice and levels were not restored by any treatment. Blood transfusion prevented the aggravation of the breakdown of blood brain barrier after artemether treatment and decreased spleen congestion without affecting splenic lymphocyte populations. Critically, blood transfusion resulted in markedly improved survival of mice with ECM (75.9% compared to 50.9% receiving artemether only). These findings indicate that whole blood transfusion can be an effective adjuvant therapy for cerebral malaria.

## Introduction

Changes in blood and blood vessels are a pathological hallmark of *Plasmodium falciparum* infection, and are particularly intense in its deadly complication, cerebral malaria (CM). Mechanical obstruction of cerebral blood vessels by sequestration of parasitized red blood cells (pRBCs) reduces cerebral blood flow and oxygen consumption^1–3^. Anemia and loss of RBC deformability impair the perfusion of various organs^4^. Severe malaria also leads to alterations of biochemical characteristics of the plasma, with depletion of plasmatic factors related with vascular health such as l-arginine^5^, haptoglobin^6^ and angiopoietin-1 (Ang-1), a critical regulator of endothelial integrity^7^. Ang-1 maintains vascular quiescence by signaling through the Tie-2 receptor^8^, whereas Ang-2, stored in Weibel-Palade bodies, can be rapidly released upon endothelial activation and displace Ang-1, sensitizing the endothelium to low concentrations of inflammatory cytokines such as TNF^8^. Indeed, Ang-2 levels are elevated in severe malaria^9^ and have been associated with CM retinopathy^10^, and blood-retinal breakdown associated with death or sequelae in pediatric CM^11^. Together with coagulation disorders like platelet activation and thrombocytopenia^12,13^, these changes disturb endothelial quiescence, leading to vascular dysfunction, impaired cerebral perfusion, acidosis^14^ and breakdown of the blood–brain barrier (BBB)^15^.

Whole blood transfusion is a practice already adopted in the adjuvant treatment of patients with severe malarial anemia^16^. In areas of high malaria endemicity, the World Health Organization (WHO) recommends blood transfusion when the hemoglobin concentration is less than 4 g/dL, this threshold is increased to 6 g/dL in case anemia is accompanied by acidosis, impaired consciousness, shock, or parasitaemia greater than 20%^17,18^. Recently, Ackerman and colleagues have shown that whole blood transfusion was associated with improved survival in children with severe falciparum malaria, and patients with impaired consciousness and hyperlactatemia benefited from transfusion even at moderate levels of anemia^19,20^.

In the present study, a well-characterized and commonly used experimental model for cerebral malaria (ECM), C57BL/6 mice infected with *P. berghei* ANKA (PbA)^21,22^, was used to investigate the effects of whole blood transfusion as adjunctive therapy to artemether in the late stages of the disease. This experimental model shows a number of similarities with human CM as well as some differences. The pros and cons of this model have been discussed^21–24^. A major difference is that a hallmark of human CM is sequestration of *Plasmodium falciparum*-infected erythrocytes in the brain post-capillary venules, resulting in vascular blockage and impaired perfusion^25,26^. In ECM, leukocyte adhesion with vascular plugging of cerebral vessels is the common pathological feature of the neurological syndrome, but *P. berghei*-infected erythrocyte accumulation in the brain has been documented and a recent study showed that *P. berghei*-infected erythrocytes are trapped in brain capillaries and contribute to impaired cerebral blood flow^27–29^. In both human and experimental CM, cerebrovascular blockage and severe vasculopathy occur, making this model appropriate to investigate interventions intended to restore vascular function, cerebral blood flow and cerebral oxygenation. Indeed, in this study whole blood transfusion showed a marked benefit on survival and on parameters associated with vascular integrity in ECM.

## Material and methods

### Animals, parasites and infection

Eight-to-ten-week-old female C57BL/6 mice (16-20 g, Fiocruz’s Institute of Science and Technology in Biomodels—ICTB-Fiocruz) were infected intraperitoneally (IP) with 1 × 10^6^ PbA (MR4 number: MRA-865) pRBCs and parasitemia checked by microscopy or flow cytometry. Hypothermia (rectal temperature between 31 and 36 °C) was used for defining late-stage ECM and as the objective criterion for treatment on day 6 post-infection^30^. Thermocouple probe (Oakton® Acorn TM; Oakton Instruments, IL, USA) was used to measure rectal temperature of mice on day 6 post-infection. All methods were performed in accordance with the relevant guidelines and regulations and the Fiocruz Animal Welfare Committee approved the experiments (license number L-037/21). The ARRIVE guidelines were taken in consideration while designing and performing experiments.

### Treatments

Two preliminary experiments were performed to determine the conditions for the main experiments. First, an experiment was performed to establish the effect of artemether and artemether plus whole blood transfusion on hematocrit in mice with late-stage ECM. Transfusion of whole blood in mice poses a substantial challenge. The typical whole blood transfusion in humans is made with an amount of 20 mL/kg, which in our mice would translate approximately to a volume of 400 μL. Transferring this amount of blood to mice, in particular mice with late-stage ECM, which present with vasoconstriction and vascular plugging by leukocyte, is even more challenging. Therefore, we followed a protocol for whole blood transfusion by means of IP injection, as previously described^31^. Following this experiment, a decision was made to use half the volume (200 μL) of whole blood for the main experiments (see “Results”). A second (survival) preliminary experiment was then conducted with three arms to define a suitable control for whole blood (200 μL) transfusion: (*i*) artemether only; (*ii*) artemether plus 200 μL of saline and; (*iii*) artemether plus 200 μL of plasma (obtained from healthy C57BL/6 mice). Artemether only showed the best outcome and was used therefore for the main experiments (see Supplementary Data).

For the main experiments, on day 6 post infection, hypothermic mice (31–36 °C) were equally and randomly distributed in two groups (ECM treated with artemether only and ECM treated with artemether plus 200 μL of whole blood transfusion). Mice received artemether (Artesiane, a kind gift of Dafra Pharma, Turnhout, Belgium) IP at 20 mg/kg as previously defined^30^. Blood was collected by cardiac puncture from a number of healthy C57BL/6 mice in sodium heparin, pooled and intraperitoneally administered (200 μL). The time elapsed between blood collection, pooling and administration to sick mice was kept below 30 min. Uninfected mice and mice with ECM, untreated, were used as controls. In the survival experiments, mice with late-stage ECM were treated with artemether with or without 200 μL of whole blood and, in the subsequent days, they received artemether only daily for another 4 days. After last artemether dose, mice were followed for 7 days before being euthanized with pentobarbital.

### Blood sample collection and analysis

Mice with ECM treated with artemether only or artemether + whole blood had their blood collected after 6 h and 24 h post treatment for hematological and biochemical analysis. Blood from uninfected, healthy mice and from mice with ECM, untreated, were used as controls. Blood was drawn by cardiac puncture and 300 μL were transferred to EDTA-coated microtubes and analyzed for hematological components, including hematocrit and platelet counts, using a pocH-100i automated hematology analyzer (Sysmex) at the Institute of Science and Technology in Biomodels (ICTB-Fiocruz). For plasma components analysis, blood was collected in heparinized tubes and centrifuged for 6 min at 6000 rpm. Plasma was collected and aliquots were made and stored at − 20 °C until needed. Spleen tissues were removed, weighed, and then processed for further analysis.

### Determination of concentrations of plasma components by enzyme-linked immunosorbent assays (ELISA)

Plasma samples were thawed and diluted to measure concentration of Ang-1 and Ang-2, using mouse Ang-1 and Ang-2 Picokine ELISA kits (Boster), or to measure mouse haptoglobin (Duoset). All ELISAs were performed according to the manufacturer’s instructions.

### Evans blue dye for blood–brain barrier permeability assay

The procedure was performed as previously described^32^. Six hours after treatment, mice were anesthetized with urethane (2 mg/g ip) with final volume 100 µL per animal. 2% solution of Evans blue dye (Sigma) in PBS 1X with final volume 150 µL was intravenously injected through orbital sinus. After 1 h of dye circulation animals were euthanized and perfused transcardially with 10 mL of ice-cold saline. Later, the brain was harvested and incubated for 48 h at 37 °C in 3 mL of 99.5% formamide (Sigma). The same procedure was done with uninfected controls, and 100 μL of formamide from each brain was then collected and absorbance measured at 620 nm. The amount of Evans blue extracted was calculated using a standard curve ranging from 1285 to 1.25 μg/mL.

### Spleen processing and immunophenotyping

Animals were submitted to cardiac perfusion with 10 mL of cold PBS. Spleens were removed, weighed and mechanically dissociated, single cell suspensions were treated with lysis buffer (Sigma) and splenocytes counted with a hematocytometer. Approximately 1 × 10^6^ spleen cells in PBS containing 5% FCS were incubated with an anti-Fc-γ III/II (CD16/32) receptor Ab (2,4G2, BD Biosciences) and pool of fluorochrome-conjugated antibodies. The following antibodies were used: PE anti-mouse TCRβ chain (H57-597, BD); APC anti-mouse CD45R/B220 (RA3-6B2, BD); APC-H7 anti-mouse CD4 (GK1.5, BD); PE-Cy7 anti-mouse CD8a (53–6.7, BD) and Percp-Cy5.5 anti-mouse CD11b (M1/70, eBioscience). Cells were incubated for 30 min at 4 °C and protected from light, according the manufacturers’ instructions. Samples were collected using a FACS CANTO II flow cytometer (BD Biosciences). Data analysis was performed using the FlowJo 10.0 program (BD Biosciences).

### Statistical analysis

All experiments were repeated at least once. Data were analyzed using a statistical software package (GraphPad Prism 7.0, La Jolla, CA). Shapiro–Wilk test was used to check distribution among the tested groups. Data are reported as mean ± standard deviation, where values of p < 0.05 were considered significant. Comparisons between 2 groups were performed using Student t-test and Mann–Whitney test, and multiple groups were compared using One-way ANOVA. For survival analysis, the Mantel-Cox log-rank test was used.

## Results

### Preliminary evaluation of whole blood transfusion on hematocrit and of saline or plasma infusion on survival in ECM

Preliminary experiments were performed in order to define the feasibility of blood transfusion via intraperitoneal injection, as described^31^, and to define a suitable treatment to compare with the performance of artemether plus whole blood transfusion in late-stage ECM. As shown in Supplemental Fig. 1A, intraperitoneal injection of 400 μL of whole blood restored hematocrit levels. However, 400 μL (20 mg/kg) is suitable for transfusion in scenarios of severe anemia, and since mice with late-stage ECM showed only mild to moderate decreases in hematocrit at the time of treatment, all the experiments were henceforth performed with 200 μL of whole blood to avoid potential deleterious effects of overtransfusion. Overtransfusion occurs when hematocrit/hemoglobin levels after transfusion of a given volume of blood exceeds the target levels, and this is known to increase the risk of death^33^. Supplemental Fig. 1B shows that artemether only was an adequate control for the experiments compared to artemether plus plasma or saline, with a better performance in survival. Indeed, addition of saline actually led to a worse outcome, with all mice dying in 24 h.

### Whole blood transfusion prevents the post-artemether decrease in hematocrit in ECM

**Figure 1:**
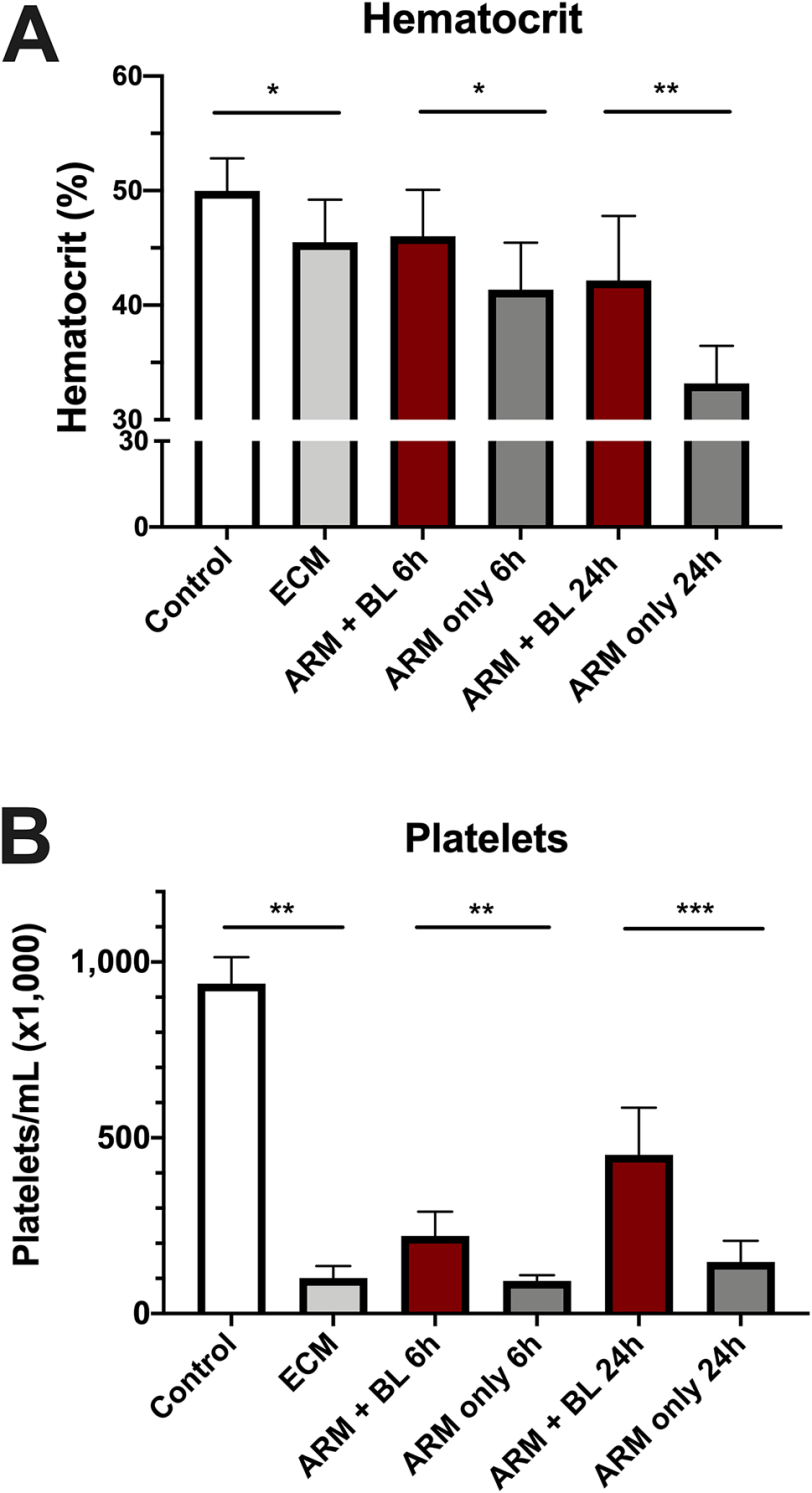
Effect of artemether treatment with and without 200 μL of whole blood on hematocrit level and platelet counts. *Plasmodium-berghei* ANKA-infected mice showing signs of ECM on day 6 of infection (n = 6–11 per group) received artemether (ARM) 20 mg/kg (20 μL) given IP, and mice in one of the groups also received 200 μL of whole blood (BL) also given IP. (**A**) Hematocrit: mice with ECM before treatment showed a mean 9% decrease in hematocrit in relation to uninfected controls (45.5 ± 3.72% versus 50.0 ± 2.83%, P = 0.0345). Treatment with ARM led to further decreases in hematocrit after 6 and 24 h (41.4 ± 4.10% and 33.2 ± 3.72%). Whole blood transfusion given together with ARM prevented the decrease in hematocrit at 6 h (ARM + BL: 46.0 ± 4.06%; ARM only: 41.4 ± 4.10%, P = 0.0352) and provided substantial protection for the strong decrease in hematocrit at 24 h (ARM-BL: 42.2 ± 5.63%; ARM only: 33.2 ± 3.72%, P = 0.0015, versus). (**B**) Platelet count: platelet count was drastically reduced by nearly 90% in mice with ECM compared to uninfected controls (101 ± 33.9 versus 938 ± 75.2, P = 0.0012). Treatment with ARM only did not change platelet levels within 6 h and 24 h. Whole blood transfusion given together with ARM led to significant recoveries in platelet counts compared to ARM only-treated mice at 6 h (221 ± 68.8 versus 93 ± 16.5, P = 0.0061) and 24 h (451 ± 134.5 versus 147 ± 59.9, P = 0.0004). Data are shown as mean ± standard deviation and Student t-test was performed for statistical analyses comparing two groups.

Mice with ECM showed a drop in hematocrit (45.5 ± 3.72%) compared to uninfected controls (50.0 ± 2.83%) (Fig. 1A). ECM mice treated with artemether alone showed a post-treatment decrease in hematocrit, reaching 41.4 ± 4.1% at 6 h and 33.2 ± 3.27% at 24 h after treatment. In contrast, ECM mice that received artemether plus 200μL of whole blood showed a preservation of hematocrit levels at 6 h (46.0 ± 4.06%) and at 24 h (42.2 ± 5.63%) (Fig. 1A).

### Whole blood transfusion partially corrects thrombocytopenia in ECM

During ECM, platelet counts (per μL of blood) fell by nearly 90% (101 ± 33.9 compared to 938 ± 75.2 of uninfected controls). Artemether treatment did not improve the platelet count at 6 h (92.8 ± 16.5) nor at 24 h (147.0 ± 60.0) post-treatment (Fig. 1B). The combination, however, of artemether treatment plus whole blood raised platelet counts by twofold at 6 h (221.0 ± 68.9) and threefold at 24 h (451.4 ± 134.5) compared to artemether-only treated mice (Fig. 1B).

### Blood transfusion decreases plasma Ang-2 levels at 24 h in ECM

**Figure 2:**
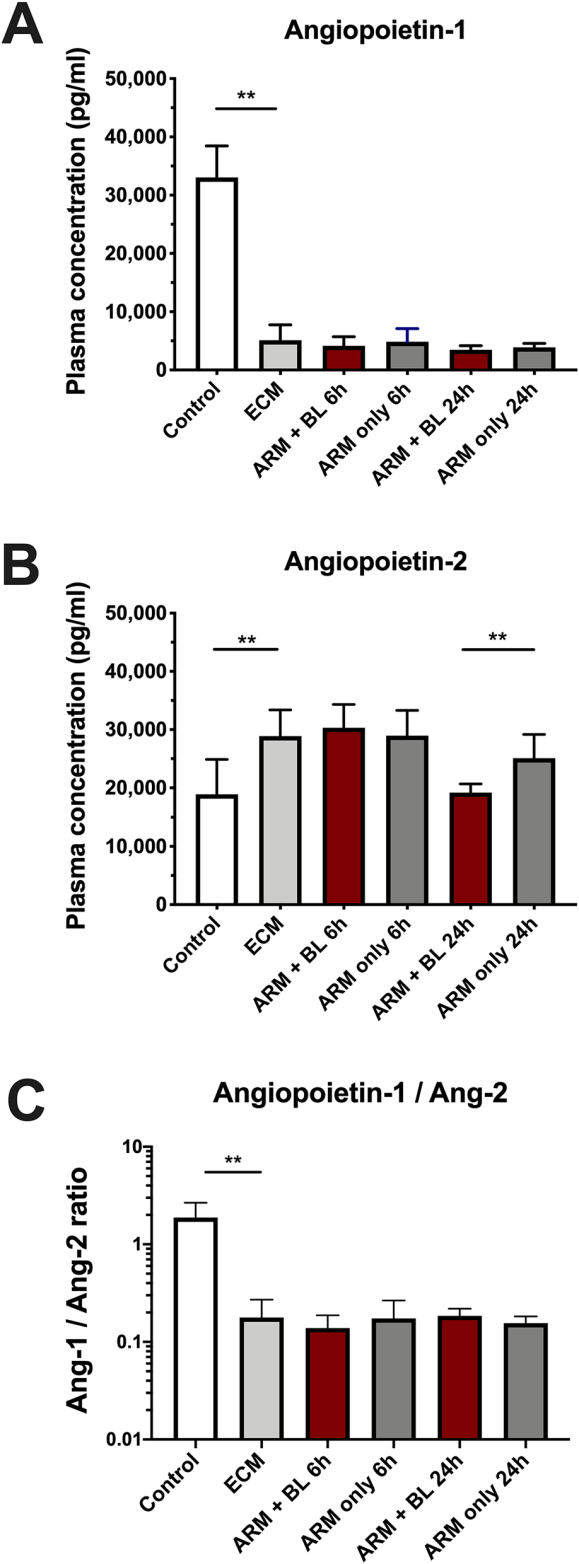
Plasma levels of Angiopoietin-1 and Ang-2 in mice with ECM treated with artemether with or without blood transfusion. Angiopoietins were assessed using ELISA (*n* = 8 mice per group). Levels of Ang-1 (**A**) were decreased by 85% in mice with ECM in relation to uninfected controls (P < 0.0001). Treatment with ARM only or ARM + BL did not change Ang-1 levels within 24 h. Levels of Ang-2 (**B**) were elevated by a mean 52% in mice with ECM in relation to uninfected controls (P = 0.0038). With blood transfusion after 24 h, concentration of Ang-2 decreased back to normal levels, whereas in ARM only treated mice levels remained high (P = 0.0090). The ratio of Ang-1 to Ang-2 (**C**) was decreased tenfold in mice with ECM compared to uninfected controls (P = 0.0002) and was not changed by ARM only or ARM + BL treatments within 24 h. Data are shown as mean ± standard deviation and Student t-test was performed for statistical analyses comparing two groups.

Mice with ECM showed very low levels of Ang-1 (5106 ± 2647 pg/mL; uninfected: 33,043 ± 5410 pg/mL) (Fig. 2A). Ang-1 levels did not recover after artemether treatment whether combined with blood transfusion or not. In contrast, Ang-2 increased to higher levels than normal from 18,887 ± 5982 to 28,882 ± 4489 pg/mL and remained high at 25,085 ± 4085 pg/mL 24 h after artemether-only treatment (Fig. 2B). Blood transfusion reversed Ang-2 levels back to normal, to 19,169 ± 1507 pg/mL after 24 h. Despite resolution of elevated Ang-2 levels after blood transfusion, the Ang-1 to Ang-2 ratios remained low (Fig. 2C).

### Haptoglobin levels, blood–brain barrier integrity and spleen weight in ECM

**Figure 3:**
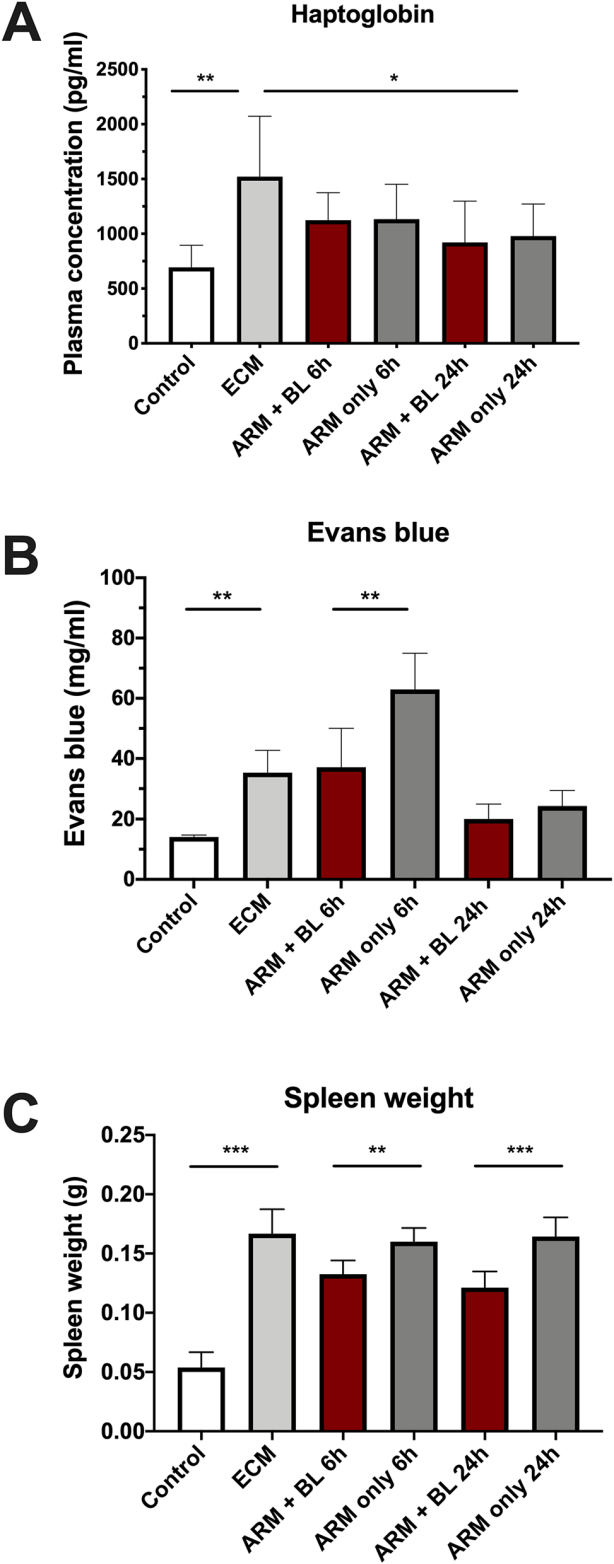
Plasma haptoglobin levels, blood–brain barrier permeability and spleen weight in mice with ECM treated with artemether with or without blood transfusion. (**A**) Plasma haptoglobin was evaluated using ELISA (*n* = 8 mice per group). Mice with ECM showed a twofold increase in plasma haptoglobin compared to uninfected control (P = 0.0074). The levels of haptoglobin were not affected by either treatment at 6 h. At 24 h, levels of haptoglobin had decreased in treated animals of both groups, being significantly different from ECM mice before treatment (P = 0.0480) and showing no significant differences in relation to uninfected control mice. (**B**) Permeability of blood brain barrier (BBB) was quantified by Evans blue assay. Mice with ECM showed increased permeability of the BBB in relation to uninfected controls (P = 0.0005). ECM mice treated with ARM only showed further increased permeability 6 h after treatment, but this increase was prevented in mice treated with ARM + BL. Indeed, permeability was significantly higher in ARM only-treated mice compared with mice treated with ARM + BL at 6 h (P = 0.0263). At 24 h, permeability decreased in both groups and was lower than in ECM mice before treatment (ARM only: P = 0.0065; ARM + BL: P = 0.0006), but was still higher than in uninfected controls (ARM only: P = 0.0032; ARM + BL: P = 0.0371). (**C**) Spleen weight: mice with ECM showed splenomegaly, with spleen weight increased more than threefold in relation to uninfected controls (P < 0.0001). Treatment with ARM only did not change spleen weight within 6 or 24 h. However, treatment with ARM + BL resulted in significant decreases in spleen weight at 6 h (P = 0.0019 and P = 0.0005 in relation to ECM mice before treatment and ARM only-treated mice, respectively) and 24 h (P = 0.0003 and P < 0.0001 in relation to ECM mice before treatment and ARM only-treated mice, respectively). However, spleen weight of ARM + BL-treated mice remained higher than uninfected controls 6 and 24 h (P < 0.0001) after treatment. Data are shown as mean ± standard deviation and Student t-test was performed for statistical analyses comparing two groups.

Figure 3A depicts the rise in plasma haptoglobin in mice with ECM (1,521 ± 525 pg/mL) compared to healthy controls (693 ± 202 pg/mL). Artemether treatment was followed by a substantial decrease in plasma haptoglobin levels (979 ± 292 pg/mL) after 24 h, and blood transfusion did not alter this decrease (921 ± 376 pg/mL).

Mice with ECM showed increased Evans blue dye leakage (35.3 ± 7.44 mg/mL; uninfected: 14.1 ± 0.66 mg/mL) (Fig. 3B). BBB integrity worsened 6 h after treatment with artemether alone (63.0 ± 12.0 mg/mL), which was prevented by whole blood transfusion (37.1 ± 13.0 mg/mL). Twenty-four hours after treatment, BBB integrity recovered in both treatment groups compared to untreated ECM mice or at 6 h post-treatment. Mice with ECM showed increased spleen weight, which did not improve 6 or 24 h after artemether-only treatment, but mice treated with artemether plus whole blood had decreased spleen weight at 6 and 24 h after treatment (Fig. 3C).

### Splenic leukocyte populations in ECM before and after treatment

**Figure 4:**
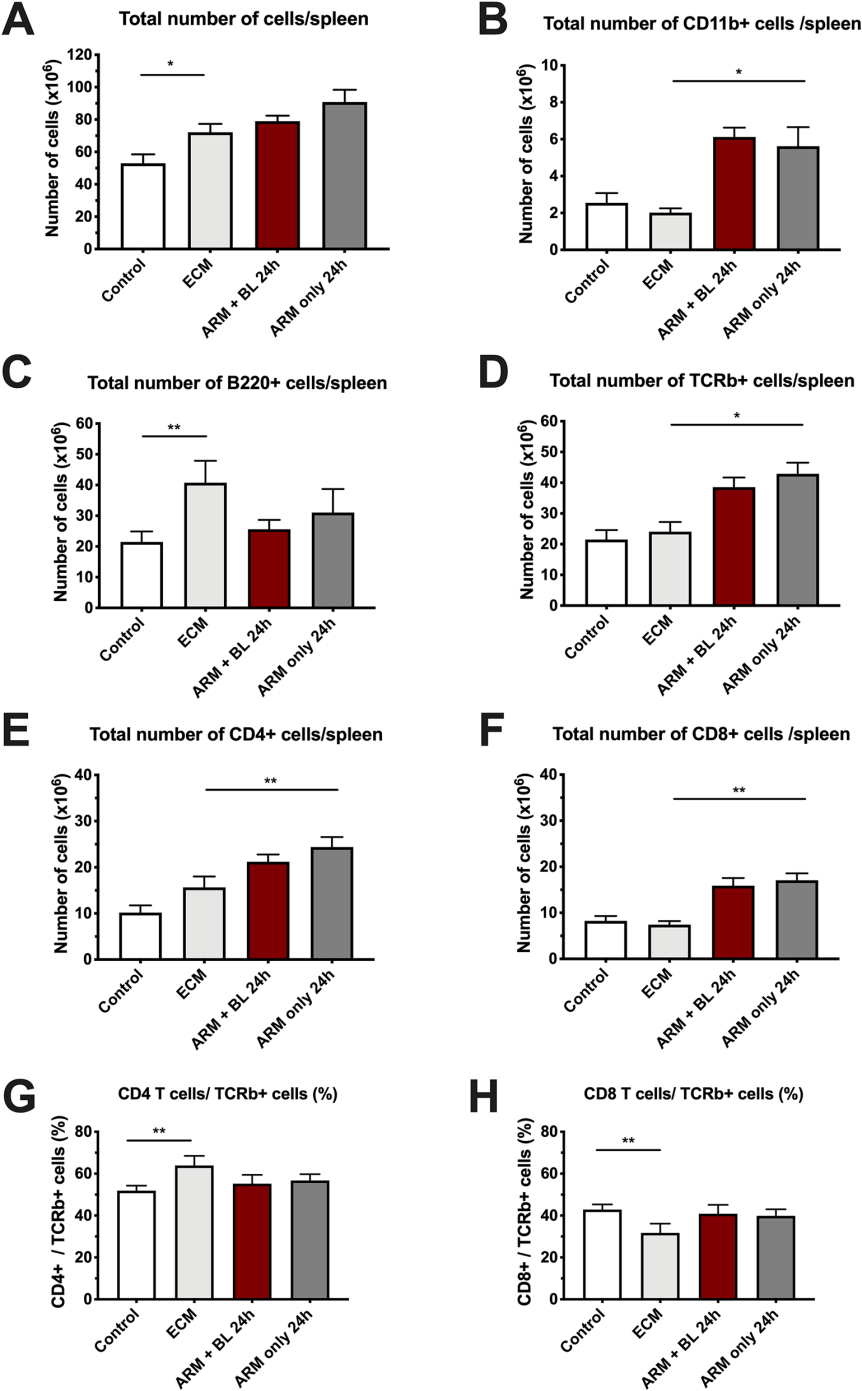
Effect of artemether treatment with and without blood transfusion on splenocyte populations. (**A**) Mice with ECM showed a mean 36% increase in the total number of splenocytes in relation to uninfected controls (P = 0.0323), which was not significantly affected by treatments after 24 h. (**B**) The number of myeloid (CD11b + cells) were not changed in mice with ECM compared to uninfected controls, but treatment with artemether, with or without blood transfusion, led to a marked increase in this population after 24 h (P < 0.0001 and P = 0.0038, respectively, compared to mice with ECM untreated). (**C**) Mice with ECM showed a marked (90%) increase in B cells (B220+) in relation to uninfected controls (P = 0.0002); 24 h after treatment, the number of B cells returned to normal levels with either treatment. (**D**) The number of T cells (TCRβ + cells) in mice with ECM was not different from that observed in uninfected controls (P = 0.5853), but numbers increased 60–78% after ARM (P = 0.0023) or ARM + BL (P = 0.0077) treatments, compared to mice with ECM untreated. (**E**) and (**F**) The effect on CD4+ and CD8+ populations was similar to that observed in TCRβ+ cells. (**G**) and (**H**) When CD4+ and CD8+ cells were analyzed in relation to their relative proportions in relation to TCRβ+ cells, an increase in CD4+ and a decrease in CD8+ cells was observed in mice with ECM, compared to uninfected controls or with ECM mice treated with artemether or artemether plus whole blood (P = 0.0004 for CD4+ cells and P = 0.0005 for CD8+ cells); these changes led to an increase in the CD4/CD8 ratio in mice with ECM untreated. Data are shown as mean ± standard deviation and Student t-test was performed for statistical analyses comparing two groups.

Data regarding splenocyte populations are shown in Fig. 4A–H. The increase in spleen weight in mice with ECM was paralleled by an increase in total number of splenocytes, which was not affected by the antimalarial treatment. The number of splenic myeloid cells was not changed in ECM mice compared to uninfected controls, but artemether treatment induced an increase in CD11b + cells. Mice with ECM showed increased numbers of splenic B cells, which return to normal 24 h after artemether treatment. The numbers of T cells and subsets (CD4+ and CD8+) did not differ in mice with ECM compared with uninfected mice. However, the relative balance was changed, with an increase in CD4+ and a decrease in CD8+ T cells. Upon treatment with artemether, the numbers and percentage of T cells and subsets increased compared to ECM mice before treatment, and the balance between CD4+ and CD8+ cells was restored. In all cases, blood transfusion had no effect on the spleen cell populations compared to artemether alone.

### Blood transfusion increases survival in artemether-treated ECM

**Figure 5:**
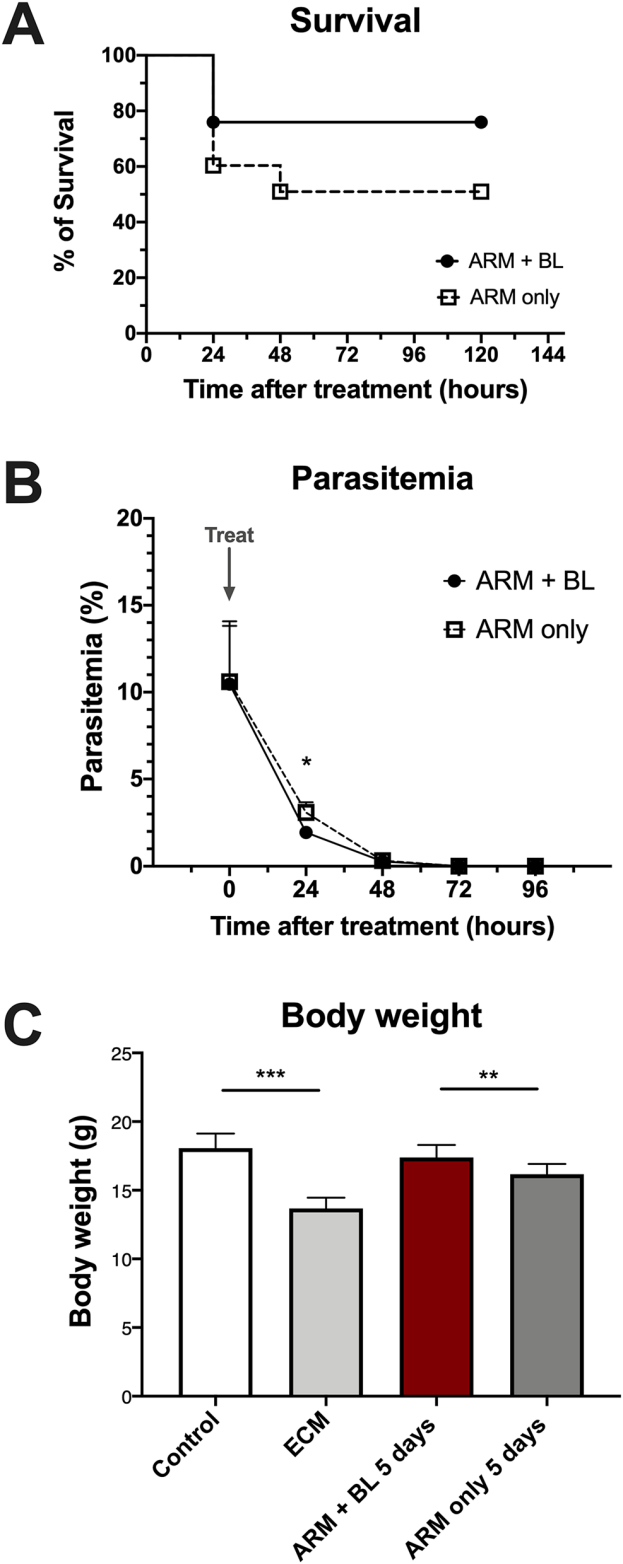
Effect of artemether treatment with and without blood transfusion on survival, parasitemia and body weight of mice with ECM. (**A**) Survival: mice with ECM treated with ARM only showed a survival rate of 50.9% (n = 53). Whole blood given as adjunctive therapy to ARM resulted in significant increase in survival to 75.9% (n = 54; P = 0.0085). Four survival experiments where conducted and results pooled. (**B**) Parasitemia: treatment with artemether, with or without whole blood transfusion, led to a marked decrease in parasitemia in 24 h. At the 24-h timepoint, parasitemia was higher in mice treated with ARM only compared to ARM + BL (3.10 ± 0.57% versus 1.94 ± 0.25%; P = 0.0046). (**C**) Body weight: mice with ECM showed a mean 24% decrease in body weight compared to uninfected controls (13.7 ± 0.79 g versus 18.1 ± 1.05 g, P < 0.0001). After 5 days of treatment, mice that received ARM + BL in the first day showed body weight (17.4 ± 0.91 g) higher than mice that received ARM only (16.2 ± 0.75 g, P = 0.0040) and not different from uninfected controls (P = 0.1391). For survival, four separate studies were performed, the results combined and log-rank test was performed for statistical analysis. For parasitemia and body weight, data are shown as mean ± standard deviation and Student t-test was performed for statistical analyses comparing two groups.

Groups of mice presenting ECM were treated with artemether (20 mg/kg) alone or combined with 200 μL of whole blood administered IP. Adjuvant therapy with blood transfusion resulted in a marked improvement of survival (75.9% versus 50.9% in the group of mice treated with artemether-only) (Fig. 5A). There was a sharp decrease of parasitemia 24 h after treatment, and at this timepoint parasitemia was lower in mice treated with artemether plus whole blood (1.94 ± 0.25%) than in mice treated with artemether only (3.10 ± 0.57%) (Fig. 5B). This difference was not observed in the subsequent timepoints. Mice with ECM showed decreased body weight (13.7 ± 0.79 g) compared to uninfected controls (18.1 ± 1.05 g) (Fig. 5C). Even after 5 days of artemether-only treatment the body weight did not fully recover (16.2 ± 0.75 g), but transfusion of whole blood in the first day of treatment with artemether led to a faster recovery of the body weight (17.4 ± 0.91 g), which was not different from uninfected controls.

## Discussion

The high lethality and post treatment neurological sequalae in patients with cerebral malaria demand new adjuvant therapies. The main finding of the present study is that whole blood transfusion resulted in substantial improvement of survival of mice with late-stage ECM treated with artemether.

Mice with ECM showed only mild to moderate (~ 10%) decrease in hematocrit. However, artemether treatment resulted in further marked drops in hematocrit after 6 and 24 h, a phenomenon also reported in human malaria^34^, especially in non-immune travelers with hyperparasitemia^35^. The fact that mice with ECM show parasitemia over 10% and parasites are rapidly killed by artemether may help to explain the rapid decrease in hematocrit following treatment.

Transfusion of 200 μL of whole blood to mice with ECM resulted in improved hematocrit at both 6 and 24 h, whereas saline had the opposite effect, and all mice died in 24 h. This worse outcome may reflect similar findings in human severe malaria, where fluid resuscitation did not improve and actually worsened patient outcomes^55–57^. Although the findings in the experimental CM model cannot be directly extrapolated for the human disease, it is expected that maintenance of the hematocrit strengthens the oxygen-carrying capacity of the blood, which in a setting of cerebral ischemia may be a critical advantage for the patient. Fresh RBCs also improve the hemorheological properties, which is known to be deteriorated in severe malaria infections^36,37^. And finally, lower hematocrit results in decreased vascular wall shear stress^38^, with decreased eNOS activity, which leads to worsened endothelial function^39^. Therefore, increasing hematocrit through whole blood transfusion should help restore endothelial function^40,41^, help clear vessels blocked by parasitized erythrocytes and inflammatory cells, increasing tissue perfusion and decreasing acidosis^19,42,43^ as well as immune cell-mediated endothelial damage. The effects on endothelial function are supported by the observation that blood transfusion prevented worsening of BBB breakdown and restored Ang-2 levels. Indeed, decreased levels of Ang-1 and increased levels of Ang-2, disturbing endothelial quiescence with loss of vascular health^7,8^, have been associated with pediatric severe malaria^10,44,45^. Since Ang-2 has been proposed as a risk factor for cognitive injury in pediatric CM, this finding is of critical importance, indicating that fresh blood counteracts the ECM-related inflammation and vascular insult. These findings are in line with data showing that interventions that counteract vascular dysfunction and inflammation are beneficial in ECM^32,40,41,46–48^. It is noteworthy, however, that the improvement in Ang-2 levels by transfusion was observed at 24 but not at 6 h, indicating that the intervention takes time to show benefit on this parameter. Since in human CM most deaths occur in the first 24–48 h after hospitalization, the relevance of this finding for clinical disease still needs to be established.

Thrombocytopenia is also one of the risk factors for mortality in African children with falciparum malaria^49^. Whole blood transfusion induced significant upturn in circulating platelets in mice with ECM. The availability of fresh, quiescent platelets, could help restoring the normality of the coagulation system without the deleterious inflammatory actions of activated platelets^50^. This effect of partially restoring the platelet counts in 24 h cannot be ascribed only to a passive, repository effect due to the platelets present in the limited amount of transfused blood. Therefore, it is apparent that blood transfusion stimulates the body to actively respond, increasing platelet production. Other effects such as improved BBB response and decreased splenic congestion also support an active modulatory, rather than just repository, effect of blood transfusion.

Acute and severe hemolysis usually leads to a consumption of haptoglobin, as seen in severe malaria^51^. In mice with ECM, anemia was only mild to moderate whereas inflammation is overwhelming, and this might help to explain why haptoglobin levels were high, since haptoglobin is an acute phase protein that increases with conditions such as inflammation and infection^52^. On the other hand, a hemolytic event might also help to explain the decrease in haptoglobin levels following artemether treatment. Blood transfusion did not seem to interfere with haptoglobin levels following artemether treatment.

In this study, 10 mL/kg (200 μL) of blood was administered, which is half the usual amount given for severe anemia, because mice with ECM showed only mild to moderate anemia and also because malaria induces splenic congestion and a sudden increase in hematocrit might exacerbate this condition. Interestingly, blood transfusion actually helped to decrease the weight of enlarged spleen in mice with ECM, suggesting that it helped to decrease congestion. It is possible that fresh red blood cells improved blood flow throughout the body, improving overall hemodynamics and decreasing the burden at the spleen. Indeed, in sickle cell disease acute splenic sequestration is treated by RBC transfusion^53^. The increase in spleen weight in mice with ECM was paralleled by an increase in the total splenocyte population. However, the magnitude of the increase in total splenocyte population was much lower than the increase in spleen weight (20% versus 200%), indicating that congestion with accumulation of blood-circulating cells was the major reason for the increased spleen weight. The vast majority of the increase in splenocyte numbers was in the B cell compartment, in line with previous findings^54^. Although there was a change in the dynamics of different splenocyte populations in mice with ECM and after treatment with artemether, blood transfusion had no effect on the outcomes of each cell population.

The benefit of blood transfusion, however, on hematological and vascular parameters in mice with ECM treated with artemether was associated with a marked improvement in survival. These findings are in line with a recent prospective multicenter observational study showing that blood transfusion improved survival of children hospitalized with severe falciparum malaria^19,20^. Blood transfusion is currently recommended by the WHO in severe malaria when hemoglobin is below 4 g/dL, or below 6 g/dL when associated with complications such as acidosis and coma. However, the authors showed that when signs of vital organ hypoperfusion are present, blood transfusion can benefit patients even at higher hemoglobin thresholds (7.7 g/dL for all severe malaria, and even higher in case of impaired consciousness or severely elevated lactate concentration)^19^. The effect of whole blood transfusion on survival in pediatric severe malaria must be evaluated in randomized controlled trials that would balance the potential benefits of transfusion against the potential risks such as infection transmission, hemolytic reactions, and circulatory overload. For patients with higher hemoglobin levels, our study in mice indicates that even the transfusion of half the usual blood volume can be of great benefit, an approach that could reduce the risk of circulatory overload.

In conclusion, the transfusion of whole blood as an adjuvant therapy in ECM showed promising results and identified an unexpected interaction between transfusion and vascular inflammation. Future clinical studies of transfusion will be necessary to evaluate the potential of this strategy as a viable, cheap and effective adjunctive therapy for cerebral malaria.

## Supplementary Information

Supplementary Information 1.

Supplementary Figure 1.
