# Supplementary material for: Whole blood transfusion improves vascular integrity and increases survival in artemether-treated experimental cerebral malaria
Source: Sci Rep. 2021 Jun 8;11:12077. doi: 10.1038/s41598-021-91499-3 (PMC8187502; doi:10.1038/s41598-021-91499-3)
Supplement: Supplementary file 1 — Supplementary Information 1. [file 41598_2021_91499_MOESM1_ESM.docx]

**Supplemental Figure 1**. **Preliminary evaluation of the effect of whole blood, plasma and saline transfusion in mice with ECM. (A)** Effect of whole blood transfusion on hematocrit: *Plasmodium-berghei* ANKA-infected mice showing signs of ECM on day 6 of infection (n = 5 per group) received artemether (ARM) 20 mg/kg (20 μL) given intraperitoneally (IP), and mice in one of the groups also received 400 μL of whole blood (BL) also given IP. At treatment, mice in both groups presented similar hematocrit levels, with mild to moderate anemia (44.0 ± 2.91 % for ARM + BL and 44.3 ± 2.51 % for ARM only; uninfected controls: 50.0 ± 2.83 %). ARM only treated mice showed progressive decreases in hematocrit 4 and 24 hours after treatment, reaching 31.6 ± 4.04 % at 24 hours (n = 3). On the other hand, mice that received 400 μL of blood transfusion together with ARM showed no further decreases in hematocrit, and actually recovered to 49.3 ± 1.06 % at 24 hours (n = 2). **(B)** Effect of saline or plasma infusion on survival: Mice with late-stage ECM received artemether (ARM) 20 mg/kg (20 μL) given IP (n = 6-13 mice per group) and were divided in three groups: *i*) received 200 μL of sterile saline, IP; *ii*) received 200 μL of plasma obtained from healthy C57BL/6 mice, IP; *iii*) received nothing additional. Mice that received artemether only showed 61% survival. In the group that received artemether + saline all mice died within 24 hours. Adding saline or plasma did not improve outcome, on the contrary.
