# Supplementary figures and images for: Whole blood transfusion improves vascular integrity and increases survival in artemether-treated experimental cerebral malaria
Source: Sci Rep. 2021 Jun 8;11:12077. doi: 10.1038/s41598-021-91499-3 (PMC8187502; doi:10.1038/s41598-021-91499-3)

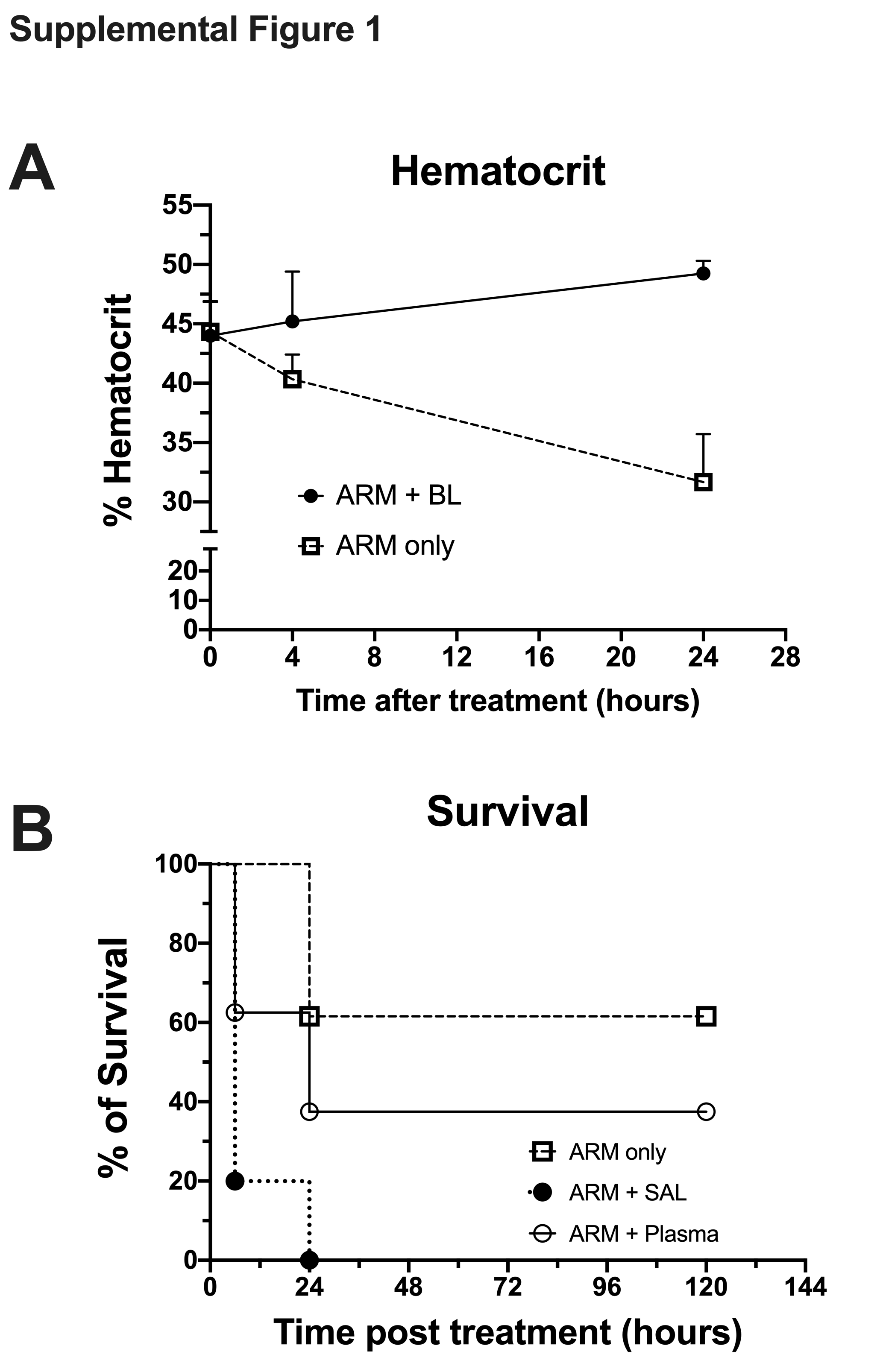

Supplement: Supplementary file 2 — Supplementary Figure 1. [file 41598_2021_91499_MOESM2_ESM.tiff]
